# Apoptotic changes and aquaporin-1 expression in the choroid plexus of cerebral malaria patients

**DOI:** 10.1186/s12936-022-04044-6

**Published:** 2022-02-12

**Authors:** Charit Srisook, Supattra Glaharn, Chuchard Punsawad, Parnpen Viriyavejakul

**Affiliations:** 1 Department of Tropical Pathology, Faculty of Tropical Medicine, Mahidol University, 420/6 Rajvithi Road, Bangkok, 10400 Thailand; 2 School of Medicine, Walailak University, 222 Thasala district, Nakhon Si Thammarat, 80161 Thailand

**Keywords:** Cerebral malaria, Choroid plexus, *P. falciparum*, Aquaporin-1, AQP-1, Caspase-3

## Abstract

**Background:**

Cerebral malaria (CM) is associated with sequestration of parasitized red blood cells (PRBCs) in the capillaries. Often, the association of CM with cerebral oedema is related with high mortality rate. Morphological changes of the choroid plexus (CP) and caspase-3 expression in CM have not been reported. In addition, limited knowledge is known regarding the role of aquaporin (AQP)-1 in CM. The present study evaluated changes in the CP, explored apoptotic changes and AQP-1 expression in CP epithelial cells (CPECs) in fatal CM patients.

**Methods:**

CP from fatal *Plasmodium falciparum* malaria patients (5 non-CM [NCM], 16 CM) were retrieved and prepared for histopathological evaluation. Caspase-3 and AQP-1 expressions in CPECs were investigated by immunohistochemistry.

**Results:**

Histologically, apoptotic changes in CPECs were significantly observed in the CM group compared with the NCM and normal control (NC) groups (*p* < 0.05). These changes included cytoplasmic and nuclear condensation/shrinkage of CPECs and detachment of CPECs from the basement membrane. The apoptotic changes were positively correlated with caspase-3 expression in the nuclei of CPECs. In addition, AQP-1 expression in CPECs was significantly decreased in the CM group compared with the NCM and NC groups (all *p* < 0.001). A negative correlation (*r*_s_ =  − 0.450, *p* = 0.024) was documented between caspase-3 expression in the nuclei of CPECs and AQP-1.

**Conclusions:**

Apoptotic changes and altered AQP-1 expression may contribute to CPEC dysfunction and subsequently reduce cerebrospinal fluid production, affecting the water homeostasis in the brains of patients with CM.

**Supplementary Information:**

The online version contains supplementary material available at 10.1186/s12936-022-04044-6.

## Background

Cerebral malaria (CM) is the most severe complication of *Plasmodium falciparum* malaria and is a major contributor to malaria fatality. The two main hypotheses of malaria pathogenesis are cytoadherence and secretion of inflammatory cytokines. Cytoadherence of parasitized red blood cells (PRBCs) to endothelial cells (ECs) in the cerebral microvasculature can induce EC injury, apoptosis, inflammation, blood brain barrier (BBB) dysfunction, brain swelling, and intracranial hypertension [1]. Pro-inflammatory cytokines, such as tumour necrosis factor (TNF) and interleukins secreted by activated leukocytes and macrophages during malaria infection, can act as apoptotic ligands bound to death receptors and induce apoptotic pathways [2, 3]. The damage caused during infection can lead to morphological changes in various tissues and organs. Apoptosis of host cells in vital organs in *P. falciparum* malaria patients, such as in the brain [2], lungs [4], liver [5], and kidneys [6], have been documented to correlate with malaria severity. The choroid plexus (CP) is a highly structured tissue that plays an important role in the production of cerebrospinal fluid (CSF) and regulation of the blood-CSF barrier (BCSFB) [7]. CP is composed of clusters of capillaries, which are covered by epithelium, and located in the lateral, third, and fourth ventricles [8].

Histologically, CP consists of a central structure of fenestrated capillaries overlaid by a single layer of epithelium with round central nuclei known as CP epithelial cells (CPECs) [9]. An important water channel protein, aquaporin (AQP), is located on the apical surface of CPECs on the cell membrane. AQP can be found in various organs such as the kidneys, lungs, salivary glands, and brain [10]. The role of AQP-1 is to control water transport during CSF production by transporting water across the CP into the cerebral ventricles [11]. A previous report on an animal model showed that gene deletion of AQP-1 causes a 25% reduction in CSF production, resulting in lower intracranial pressure [12]. However, a recent study on an animal model has suggested that AQP-1 may also contribute to hypersecretion of CSF and post-haemorrhagic hydrocephalus [13]. Currently, the role of AQP-1 in diseases is limited, and the modulation of AQP-1 in CM is still unclear. This work was a pioneer study to evaluate the histopathological changes of the CP in CM and to investigate the expression of caspase-3 and AQP-1 in CPECs. The results of this study could document CPEC changes and the important involvement of AQP-1 in CM, which could contribute to a new insight in CM pathogenesis.

## Methods

### Specimen preparation

CP from *P. falciparum* malaria infected patients and normal control CP samples were obtained from the Department of Tropical Pathology, Faculty of Tropical Medicine, Mahidol University, Bangkok, Thailand. Samples from the CP were divided into three groups: normal control (NC, n = 5), non-CM (NCM, n = 5), and CM (n = 16). In the NC group, samples from the CP, which showed normal CPECs and blood vessels, were obtained from patients who died from accidents. The study protocol was approved by the Ethics Committee of the Faculty of Tropical Medicine, Mahidol University, Thailand (MUTM 2018-041-01 and MUTM 2018-041-02).

### Histopathology and evaluation

Samples from the CP were processed and embedded in paraffin blocks. The blocks were sectioned at 4-µm thickness for histopathology and immunohistochemistry studies. The CP sections were stained with modified haematoxylin and eosin. Histopathological features of the apoptotic changes in CPECs, PRBC sequestration, and malaria pigment/haemozoin deposition were evaluated in 10 microscopic fields under high power fields (HPF) (400×) per slide. Features of apoptotic changes were cytoplasmic and nuclear condensation/shrinkage of CPECs and detachment of CPECs from the basement membrane. Apoptotic changes and PRBC sequestration were recorded in percentages. For quantification of malaria pigment/haemozoin deposition, Image J software program (National Institute of Health, USA) was used to determine the density of malaria pigments/haemozoin in the tissue in relation to the whole HPF and presented in percentage.

### Immunohistochemistry studies of caspase-3 and AQP-1

The expressions of caspase-3 and AQP-1 were detected by immunohistochemical staining. Samples from 4-µm CP sections were placed on adhesive slides coated with poly-L-lysine. The CP sections were deparaffinized in xylene and rehydrated with graded concentrations of alcohol. Antigen retrieval from the CP was performed by a microwave technique with 0.1 M citrate buffer at pH 6.0 for 10 min. To reduce endogenous peroxidase activity, the sections were incubated with 3% hydrogen peroxide in distilled water for 10 min at room temperature. After washing in phosphate-buffered saline (PBS, pH 7.4), the non-specific binding site was blocked with normal goat serum for 30 min at room temperature. Sections were incubated overnight at 4 °C with the specific primary antibody of rabbit polyclonal antibody directed against caspase-3 (1:400; Cell Signaling Technology, USA) and AQP-1 (1:2000; Abcam, Cambridge, UK). During the ensuing days, sections were washed three times with PBS and incubated with a secondary antibody for 30 min at room temperature and reacted with an avidin–biotin complex conjugated with horseradish peroxidase (Vector Laboratories, Inc., CA, USA) and performed according to the manufacturer’s instructions. After washing with PBS, 3,3′-diaminobenzidine tetrahydrochloride was used to visualize for peroxidase activity (brown color). Sections were counterstained with haematoxylin, dehydrated, and mounted with a cover slip.

### Evaluation of immunohistochemical staining

The immunohistochemistry study was assessed based on both semi-quantitative and qualitative results as well as the distribution of caspase-3 and AQP-1 in the CP. For semi-quantitative data, each slide was evaluated in 10 different microscopic fields at 400× magnification for immunopositive cells. The percentage of immunopositive cells in each field was calculated and compared with the number of total cells. For qualitative data, the intensity of immunopositive cells was graded on a scale of 0 to 3 as follows: no staining = 0; weak positive staining = 1; moderate positive staining = 2; and strong positive staining = 3. The total score (TS) was calculated from the product of the percentage of immunopositive cells and intensity of staining [2]. For caspase-3 expression, both cytoplasmic and nuclear staining were recorded. The sections were examined in a blinded manner without prior knowledge of the patients and clinical status. Histopathology and immunohistochemistry were evaluated by two independent observes (CS and SG) who were unaware of the patients’ clinical outcomes. When inter-observer disagreement occurred, a third investigator was requested to evaluate the samples (PV).

### Statistical analysis

Data were recorded into a computer database and analysed with SPSS software version 18.0 (SPSS, USA). All quantitative data were represented as mean ± standard error of mean (SEM). The test for normality of distribution was calculated using the Kolmogorov–Smirnov Test. The independent *t*-test was used to analyse differences in clinical data between the NCM and CM groups. Comparisons of differences in clinical data (admission and last parasitaemia), histopathological changes, and caspase-3/AQP-1 expressions between groups were calculated using the Mann–Whitney U test. The correlations between the TS of caspase-3/AQP-1 expressions and histopathological changes/clinical data were analysed using Spearman’s correlation. A *p*-value of < 0.05 was considered statistically significant.

## Results

### Summary of clinical data from malaria patients

Table 1 summarizes the clinical parameters, including age, gender, febrile days, haemoglobin level, white blood cell count, and parasite count between the NCM and CM groups. Levels of parasitaemia on admission and before death were significantly higher in the CM group than in the NCM group (*p* < 0.05). The causes of death for the NCM group included co-infection with gram-negative organisms, disseminated intravascular coagulation, left-sided heart failure with severe pulmonary oedema, pneumonia, and acute respiratory distress syndrome. The time elapsed between death and sampling of the CP was between 3 and 5 h in all groups.

**Table 1:** Clinical data of the malaria patients

Parameters	Non-cerebral malaria (n = 5)	Cerebral malaria (n = 16)	*p*-value
Age (years)	43.80 ± 11.13	36.81 ± 4.95	0.524
Sex (M:F)	3:2	10:6	–
Days of fever	5.75 ± 0.94	3.87 ± 0.69	0.146
First Hb (g/dl)	9.98 ± 0.58	10.05 ± 0.64	0.948
Last Hb (g/dl)	9.18 ± 0.90	9.74 ± 0.62	0.630
First WBC (cell/mm^3^)	12,371.04 ± 4,342.15	12,458.31 ± 2,005.65	0.984
Last WBC (cell/mm^3^)	15,474.04 ± 6,748.08	15,297.46 ± 2,193.29	0.981
Parasitaemia (admission) (/µl)	132,035 ± 131,988.30	587,787.30 ± 159,714.30	0.039*
Parasitaemia (last) (/µl)	10.50 ± 10.50	294,182.90 ± 125,024.70	0.014*

^*^Significant difference of *p* < 0.05

### Histopathological changes of CP in *P. falciparum* malaria patients

**Fig. 1:**
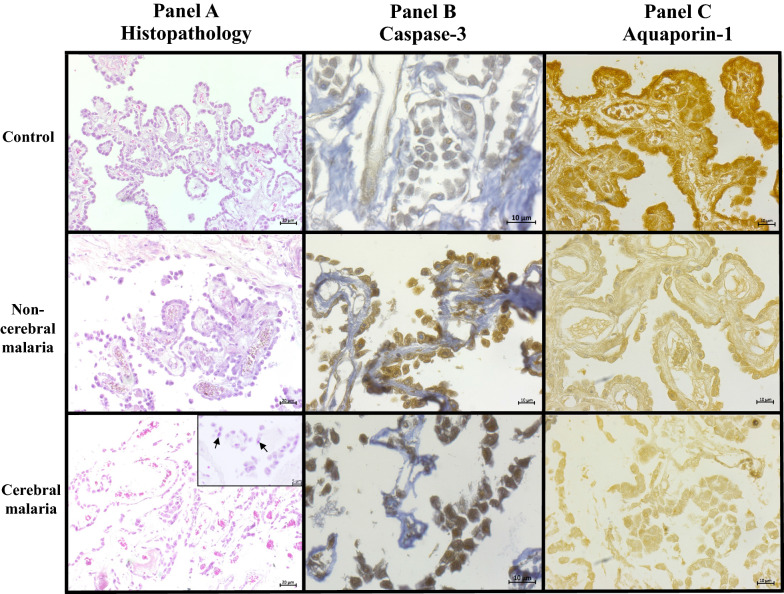
Histopathology, caspase-3, and aquaporin-1 expressions of CPECs in the normal control (NC), non-cerebral malaria (NCM), and cerebral malaria (CM) groups. In **A**, the normal CP displays a central structure of capillaries, which is overlaid by a single layer of CPECs attached to the basement membrane. The CP from the NCM group shows shrinkage of CPECs and detachment of a few CPECs. Marked nuclear condensation, CPEC shrinkage (inset, arrows), and detachment of CPECs from the basement membrane are noted in the CM group. **B** illustrates caspase-3 expression in CPECs. The NCM and CM groups display enhanced nuclear expression of caspase-3, which is significantly increased in the CM group. **C** demonstrates aquaporin (AQP)-1 expression in CPECs. The NC group shows high expression of AQP-1 in CPECs. A marked decreased in AQP-1 expression was noted in the CM group. (Images in (**A**): 200 × magnification; images in (**B**) and (**C**): 400 × magnification)

**Fig. 2:**
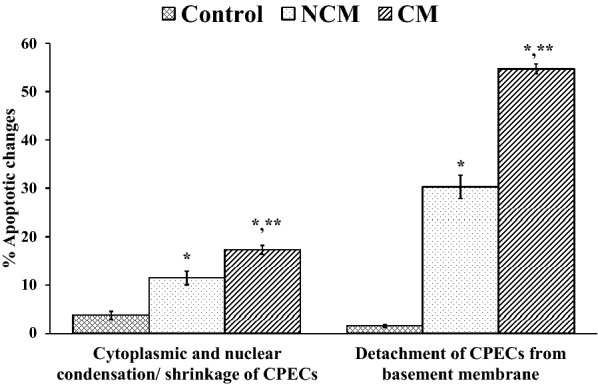
Apoptotic changes of CPECs in the normal control (NC), non-cerebral malaria (NCM), and cerebral malaria (CM) groups. *Significant difference of *p* < 0.001 compared with the NC group. **Significant difference of *p* < 0.05 compared with the NCM group. Data are presented as mean ± SEM

**Fig. 3:**
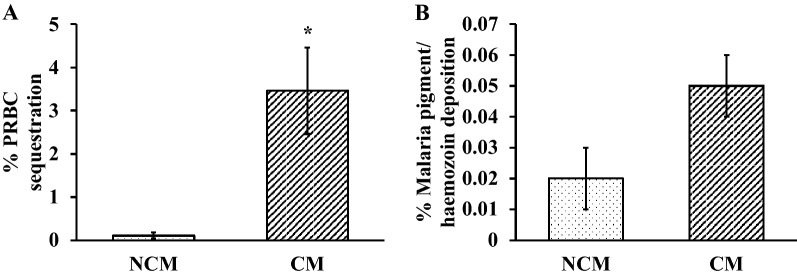
Comparative parasitized red blood cell sequestration (**A**) and malaria pigment/haemozoin deposition (**B**) in the non-cerebral malaria (NCM) and cerebral malaria (CM) groups. *Significant difference of *p* < 0.05 compared with the NCM group. Data are presented as mean ± SEM

Normal histology of the CP showed a layer of CPECs surrounding a core of capillaries. Figure 1 Panel A represents the histopathological changes of the CP in the NC, NCM, and CM groups. For the NC group, the cytoplasm stained homogeneously pink, and the nucleus appears oval, clear, and visible. The CP from the NCM group showed shrinkage of CPECs and detachment of a few CPECs from the basement membrane. Marked nuclear condensation, CPEC shrinkage, and detachment of CPECs from the basement membrane were noted in the CM group. CPECs in the NCM group showed lesser degree of apoptotic changes compared to those in the CM group. Figure 2 shows the comparative apoptotic changes of CPECs in the NC, NCM, and CM groups (data in Additional file 1: Table S1). In both malaria groups (NCM and CM), CPECs showed significant increase in apoptotic changes compared to those of the NC group (all *p* < 0.001). The CM group had the apoptotic changes, which were significantly increased compared to those of the NCM group (*p* < 0.05). Regarding PRBC sequestration in the capillaries of the CP, the CM group (3.46 ± 1.00) showed significantly higher sequestration than the NCM group (0.11 ± 0.07) (*p* = 0.002) (Fig. 3A). However, no significant difference was observed in malaria pigment/ haemozoin deposition between NCM (0.02 ± 0.01) and CM groups (0.05 ± 0.01) (*p* = 0.086) (Fig. 3B). In addition, no correlation between malaria pigment/ haemozoin deposition and apoptosis of CPECs was observed (Additional file 2: Fig. S1). The interstitium of the CP showed minimal swelling and scant inflammatory cells in all groups.

### Expression of caspase-3

Both nuclear and cytoplasmic accumulations of effector caspase-3 expression were observed in CPECs. Considering the percentage of immunopositive cells and intensity of staining, the TS for caspase-3 was compared across all groups. Figure 1 Panel B illustrates the caspase-3 expression in the CPECs in the NC, NCM and CM groups. Table 2 summarizes the TS of caspase-3 expression in the CPECs. In the NC group, cytoplasmic accumulation was significantly more prevalent compared to nuclear localization. For nuclear localization, caspase-3 expression in the CPECs was significantly highest in the CM group compared to that of the NC (*p* < 0.001) and NCM (*p* = 0.018) groups. There was a significant difference in caspase-3 cytoplasmic localization between the NC and NCM groups (*p* = 0.005). In addition, the CM group showed a significant increase in both nuclear and cytoplasmic accumulation of caspase-3 compared to those of the NC and NCM groups (all* p* < 0.001).

**Table 2:** Total score of caspase-3 in CPECs in normal control, non-cerebral malaria and cerebral malaria groups

Groups	Total score of caspase-3 (% positive cells × staining intensity)		
	Nuclear staining	Cytoplasmic staining	Nuclear and cytoplasmic staining
Normal control	5.93 ± 0.57	80.67 ± 7.37	40.07 ± 4.49
Non-cerebral malaria	45.33 ± 7.97*	60.33 ± 8.02*	47.67 ± 3.90
Cerebral malaria	67.11 ± 4.09*^,^**	75.00 ± 6.18	104.56 ± 8.65*^,^**

^*^Significant difference of *p* < 0.05 compared with NC group

**Significant difference of *p* < 0.05 compared with NCM group. Data are presented as mean ± SEM

**Fig. 4:**
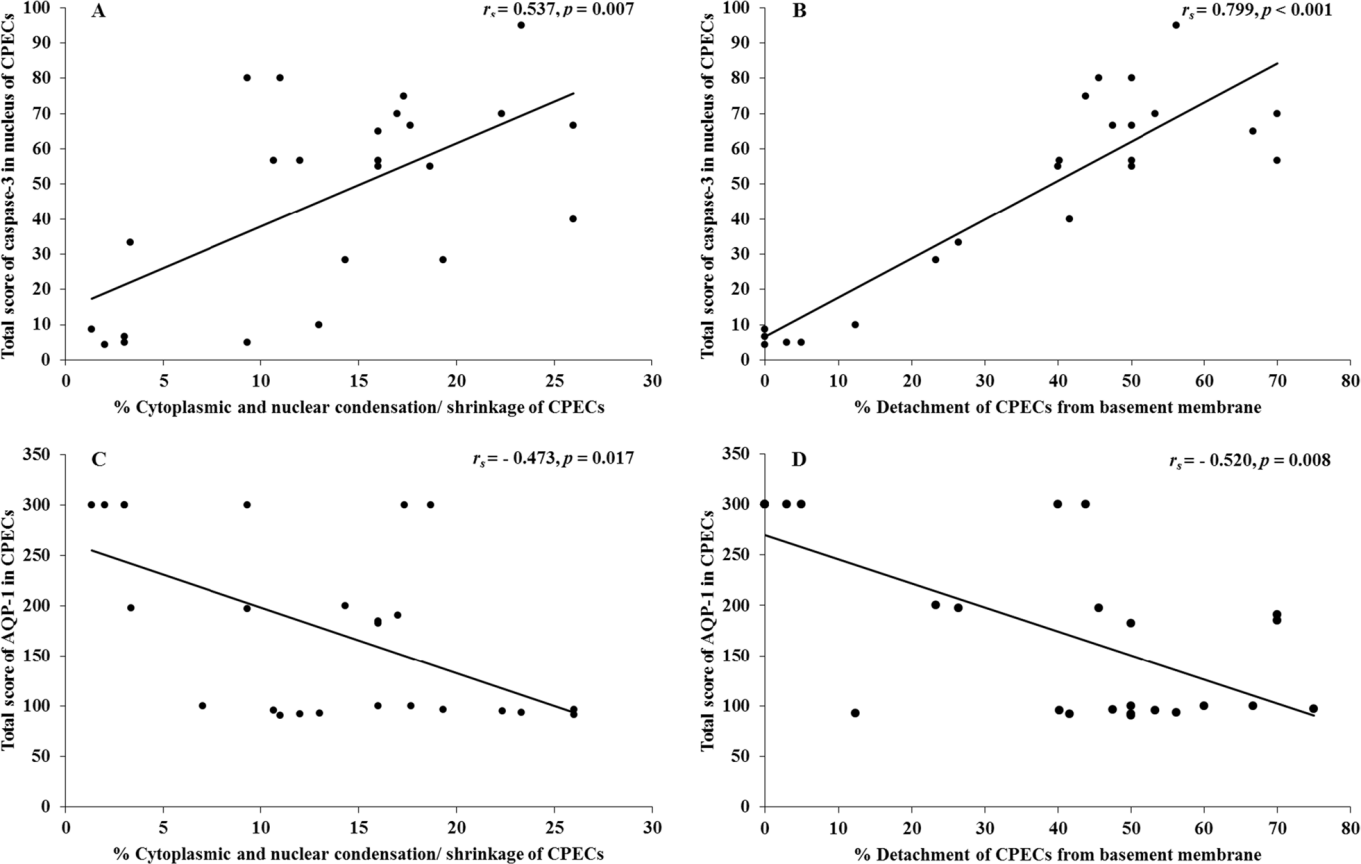
Correlations between caspase-3 (**A** and **B**) and AQP-1 (**C** and **D**) expressions and apoptotic changes in choroid plexus epithelial cells

Nuclear accumulation of caspase-3 in CPECs was positively correlated with the apoptotic changes of CPECs (cytoplasmic and nuclear condensation/ shrinkage of CPECs, *r*_s_ = 0.537, *p* = 0.007) (Fig. 4A) and degree of CPEC detachment from the basement membrane, (*r*_s_ = 0.799, *p* < 0.001) (Fig. 4B). However, cytoplasmic accumulation of caspase-3 was negatively correlated with cytoplasmic and nuclear condensation/ shrinkage of CPECs (*r*_s_ =  − 0.492, *p* = 0.015). No correlation was observed between cytoplasmic accumulation of caspase-3 and the degree of CPEC detachment from the basement membrane (*r*_s_ =  − 0.299, *p* = 0.892), PRBC sequestration (*r*_*s*_ = 0.188, *p* = 0.442), and presence of malaria pigments/haemozoin (*r*_*s*_ = 0.172, *p* = 0.469) (Additional file 3: Fig. S2).

### Expression of AQP-1

AQP-1 expression stained brown color at the apical surface and cytoplasm of CPECs Fig. 1 Panel C demonstrates the comparative immunostaining for AQP-1 in the NC, NCM, and CM groups. The quantification of AQP-1 expression in CPECs in the different groups is tabulated in Table 3. CPECs from the NC group showed full expression of AQP-1 with maximal intensity. In contrast, AQP-1 expression was significantly decreased in the CM group when compared with those of the NC and NCM groups (all *p* < 0.001). For staining intensity, moderate positive staining was observed in the NCM group, whereas weak staining was observed in the CM group. The TS of AQP-1 was negatively correlated with the apoptotic changes of CPECs, cytoplasmic and nuclear condensation/shrinkage of CPECs (*r*_s_ =  − 0.473, *p* = 0.017) and degree of CPEC detachment from the basement membrane (*r*_s_ =  − 0.520, *p* = 0.008) (Fig. 4C and D).

**Table 3:** AQP-1 expression in CPECs in normal control, non-cerebral malaria and cerebral malaria groups

Groups	AQP-1 expression (%)	Staining intensity	Total score (% positive cells staining intensity)
Normal control	100.00 ± 0.00	3.00 ± 0.00	300.00 ± 0.01
Non-cerebral malaria	98.05 ± 0.74*	2.00 ± 0.10*	197.55 ± 9.78*
Cerebral malaria	95.31 ± 0.43*^,^**	1.33 ± 0.05*^,^**	127.26 ± 5.06*^,^**

^*^Significant difference of *p* < 0.001, compared with NC

**Significant difference of *p* < 0.001, compared with NCM

**Fig. 5:**
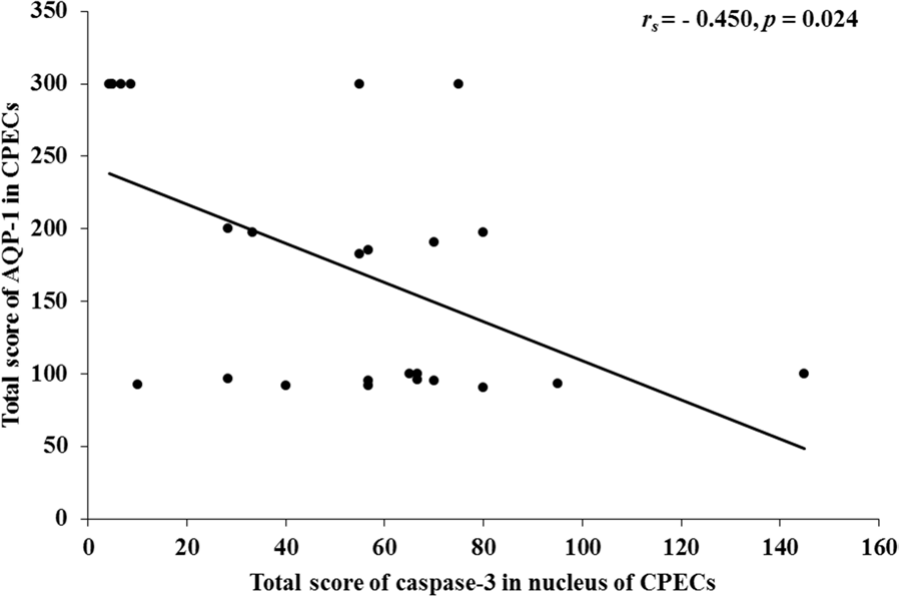
Correlations between caspase-3 and AQP-1 expressions in choroid plexus epithelial cells

In addition, AQP-1 expression was negatively correlated with caspase-3 accumulation in the nucleus of CPECs (*r*_s_ =  − 0.450, *p* = 0.024) (Fig. 5). No correlation was observed between caspase-3 expression in the cytoplasm alone or cytoplasm and nucleus and AQP-1 expression in CPECs (*r*_*s*_ =  − 0.062, *p* = 0.768; *r*_*s*_ =  − 0.349, *p* = 0.087, respectively). In addition, no correlation was observed between AQP-1 and PRBC sequestration (*r*_*s*_ = 0.091, *p* = 0.711) and presence of malaria pigments/haemozoin (*r*_*s*_ = 0.311, *p* = 0.183) (Additional file 4: Fig. S3).

## Discussion

An important process underlying CM is cytoadhesion of PRBCs to the cerebral vascular endothelium and subsequent sequestration in the microvasculature. In addition, rosette formation and agglutination can worsen microvascular obstruction, resulting in transient cerebral ischaemia. Injury to ECs from post-adhesion events and malaria pigments/haemozoin can lead to cerebral haemorrhage and nervous tissue damage [1]. The present study investigated changes in the CP, including apoptotic changes and expression of AQP-1 in CM. CP is responsible for the production of CSF, which as part of the BCSFB has an important role of preventing foreign body entry into the ventricles [7]. Generally, pathological changes to the CP include damage to three compartments, namely CPECs, the interstitium, and blood vessels [14]. The current study documented the significant apoptotic changes of CPECs in the CM group. Changes consisted of cytoplasmic and nuclear condensation/shrinkage of CPECs and detachment of CPECs from the basement membrane. Histopathological changes of apoptosis were significantly associated with the expression of caspase-3, which is the final apoptotic pathway. However, the interstitium of the CP, which is normally accessible to cells of the peripheral immune system including macrophages, showed minimal inflammatory cells in both malaria groups. A significant finding was the accumulation of caspase-3 within the nuclei, which is suggestive of CM induced pro-caspase-3 cleavage resulting in activated caspase-3 transfer to the nuclei to cleave nuclear substrates. As previously reported, characteristic apoptotic nuclear changes, such as DNA fragmentation, chromatin condensation, and nuclear disruption, have been visualized [15]. These apoptotic changes could be identified under a light microscope as condensation or shrinkage of the cytoplasm, nucleus, or the whole cell. Further to CPEC contraction, weakening of cell adhesion to the basement membrane can lead to CPEC detachment, a feature seen significantly in the CM group. In addition, PRBCs and malaria pigments/haemozoin associated with the CM group were seen scattered within the capillaries of the CP. Regarding morphology, it would be informative to measure the surface area of CPECs to establish the alteration in CPEC size across the three experimental groups.

The cytoadhesion and sequestration processes in severe malaria can affect CP circulation and eventually lead to CSF obstruction and cerebral oedema. In addition, PRBCs and malaria pigments/haemozoin within the capillaries have been documented to activate nuclear factor kappa B (NF-κB) [16]. Punsawad et al. reported that soluble mediators in sera of malaria patients can activate NF-κB in cerebral blood vessels, which resulted in apoptotic changes in ECs [2]. It can be hypothesized that PRBCs and malaria pigments/haemozoin within the capillaries of the CP can trigger apoptotic changes in CPECs. A previous study also showed that malaria pigments/haemozoin can trigger the release of matrix metalloproteinases (MMPs) from monocytes/macrophages, which could damage the BBB [17]. MMPs have been reported to not only cause the destruction of junctional proteins of ECs, such as zonula occludens-1, occludin, and claudin proteins [18], but also destroy the extracellular matrix, which could damage CPEC cell junctions and cause CPEC detachment from the basement membrane. These apoptotic changes resemble the histopathological alterations observed in the CP of the CM group. The resulting EC junctional changes and apoptosis of CPECs caused by cytoadhesion and circulating cytokines in CM could contribute to an increase in EC permeability, hence altering the BBB.

Changes in CPECs have been reported in ischaemia [19], aging [20, 21], Alzheimer’s disease [22], and in various infections [23–27]. Examples of CPEC damage in brain infections include disruption of CPECs by the Zika virus [23], SARS-CoV-2 [24], *Streptococcus suis* [25], *Trypanosoma evansi* [26], and *Leishmania chagasi* [27]. These infections resulted in the breakdown of BBB integrity [23, 24, 26], infiltration of inflammatory cells [26], and CPEC apoptosis [27]. In an animal model, an electron microscopy study of ischaemia in the CP showed acute injury and necrotic changes of CPECs. These changes include a decrease in nuclear sizes, swelling of intracellular organelles, loss of microvilli, damage of cell membranes, disruption of cell junctions, cellular necrosis, and accumulation of fibroblasts [19]. In malaria, a previous electron microscopic study of ECs in the CP showed capillary EC swelling and bulging of EC nuclei into the vascular lumen in addition to cytoadhesion of PRBCs to ECs [28]. Clinically, fatal CM is associated with profound cerebral hypoxic injury in adults, as measured by low apparent diffusion coefficient, which is likely due to sequestered PRBCs [29, 30].

TNF released during an acute malaria infection can cause damage to the CPEC barrier. Previous reports on porcine CPECs have documented that TNF induces CPEC barrier alterations as evidenced by an increase in cell permeability, DNA fragmentation, chromatin condensation, and activation of caspase-3 [31]. In addition, cytoadhesion can trigger TNF signal transduction via TNF receptors to induce death receptor-mediated apoptotic pathways and alter tight junction functions in part via MMP [32]. A significant increase in caspase-3 and -9 expressions could ultimately lead to cell death, increased cell permeability, and trigger CSF barrier dysfunction [33].

The study further investigated the expression of AQP-1 in CPECs in malaria, which demonstrated the diminished expression of AQP-1 in the CM group. AQP-1 was more enhanced at the apical membrane as compared to the basolateral membrane of CPECs, similar to a previous report [11]. In an animal model, AQP-1 expression deficiency in CPECs caused inadequate CSF production [12], which can result in abnormal fluid homeostasis in the central nervous system. Reduced AQP-1 expression can reduce CSF production, which is protective for hydrocephalus [34]. Decreased AQP-1 expression in the CP is known to be associated with cerebral ischemic oedema. A previous study on rat CP showed that within 1–24 h of an ischemic event, diminished blood flow to the CP caused necrosis of CPECs and was related to a decrease in CSF production [35]. A study on mice with lungs infected with adenovirus demonstrated that AQP-1 and -5 expressions could decrease the occurrence of pulmonary oedema by reducing vascular permeability to the lung interstitium [36]. However, a study on the lungs of patients with malaria showed that AQP-1 and -5 have no roles in decreasing cellular permeability [37]. In severe *P. falciparum* malaria, particularly CM, sequestration can lead to capillary obstruction, transient cerebral ischaemia, and a reduction in AQP-1 expression. The association between AQP-1 and caspase-3, leading to the process of apoptosis has been linked to the mitogen-activated protein kinase (MAPK) signaling pathway [38]. AQP-1 has been reported to decrease caspase-3 expression, subsequently reversing apoptotic activity via p38 and ERK1/2 in lipopolysaccharide-induced human proximal tubule cell line (HK-2 cells) [38]. In addition, a previous study on lung ischaemia reperfusion injury showed upregulation of caspase-3 and a decrease in AQP-1 expression through the p38 MAPK pathway [39].

From the present study, apoptotic changes in CPECs (histopathological changes and expression of caspase-3) were correlated with a decrease in AQP-1 expression in the CM group. Inadequate AQP-1 can lead to a reduction in CSF production, deficiency in essential CSF proteins, and accumulation of toxic substances. Consequently, CPEC damage is enhanced. In addition, CPEC changes in CM could lead to an increase in cellular permeability and possibly accumulation of fluid in the ventricles. On the contrary, the occurrence of cerebral oedema from a damaged BBB may be compensated by a reduction in AQP-1. Whether cerebral oedema in CM is prevented by a decline in AQP-1 needs further investigation.

## Conclusions

CM causes apoptotic changes to CPECs as evidenced by the morphological changes and enhanced expression of caspase-3. This study also demonstrates the functional relevance of AQP-1 expression in CPECs and may hint to a decrease in incidence of brain oedema associated with AQP-1 reduction in CM.

## Supplementary Information

**Additional file 1: Table S1.** Quantitative data of histopathological changes of the choroid plexus in *P*. *falciparum* malaria patients.

**Additional file 2: Figure S1.** No pathological correlation between malaria pigment deposition and apoptosis of CPECs (A- cytoplasmic and nuclear condensation/shrinkage of CPECs, and B- detachment of CPECs from the basement membrane).

**Additional file 3: Figure S2.** No correlation between caspase-3 and the degree of CPEC detachment from the basement membrane (A), PRBC sequestration (B), and presence of malaria pigments/haemozoin (C).

**Additional file 4: Figure S3.** No correlation between AQP-1 expression in CPECs and caspase-3 expression in cytoplasm alone (A) and cytoplasm and nucleus (B), PRBC sequestration (C), and presence of malaria pigments/ haemozoin (D).

## Data Availability

All data generated or analysed during this study are included in this published article.

## Abbreviations

AQP: Aquaporin
BBB: Blood brain barrier
BCSFB: Blood-CSF barrier
CM: Cerebral malaria
CP: Choroid plexus
CPECs: Choroid plexus epithelial cells
CSF: Cerebrospinal fluid
ECs: Endothelial cells
HPF: High power field
MAPK: Mitogen-activated protein kinase
MMPs: Matrix metalloproteinases
NC: Normal control
NCM: Non-cerebral malaria
NF-κB: Nuclear factor kappa B
*p*: Probability value
PBS: Phosphate-buffered saline
PRBCs: Parasitized red blood cells
*r*_s_: Spearman correlation coefficient
SEM: Standard error of the mean
SPSS: Statistical package for the social sciences
TNF: Tumour necrosis factor
TS: Total score
