# Supplementary material for: Apoptotic changes and aquaporin-1 expression in the choroid plexus of cerebral malaria patients
Source: Malar J. 2022 Feb 12;21:43. doi: 10.1186/s12936-022-04044-6 (PMC8841049; doi:10.1186/s12936-022-04044-6)
Supplement: Supplementary file 1 — Additional file 1: Table S1. Quantitative data of histopathological changes of the choroid plexus in P. falciparum malaria patients. [file 12936_2022_4044_MOESM1_ESM.doc]

**Apoptotic changes and aquaporin-1 expression in the choroid plexus of cerebral malaria patients**

**Supplementary information**

**Additional file 1: Table S1 Quantitative data of histopathological changes of choroid plexus in *P. falciparum* malaria patients**

| **Histopathological changes** | **Normal control** | **Non-cerebral malaria** | **Cerebral malaria** |
| --- | --- | --- | --- |
| % Cytoplasmic and nuclear condensation/ shrinkage of CPECs | 3.73 ± 0.81 | 11.47 ± 1.41* | 17.27 ± 0.91*,** |
| % Detachment of CPEC from basement membrane | 1.60 ± 0.32 | 30.29 ± 2.44* | 54.70 ± 1.02*,** |
| % PRBC sequestration | NA | 0.11 ± 0.07 | 3.46 ± 1.00** |
| % Malaria pigment/ haemozoin deposition/HPF | NA | 0.02 ± 0.01 | 0.05 ± 0.01 |

*Significant difference of *p* < 0.001 compared with NC group. **Significant difference of *p* < 0.05 compared with NCM group. Data are presented as mean ± SEM.
