# Supplementary figures and images for: Apoptotic changes and aquaporin-1 expression in the choroid plexus of cerebral malaria patients
Source: Malar J. 2022 Feb 12;21:43. doi: 10.1186/s12936-022-04044-6 (PMC8841049; doi:10.1186/s12936-022-04044-6)

## Slide 1
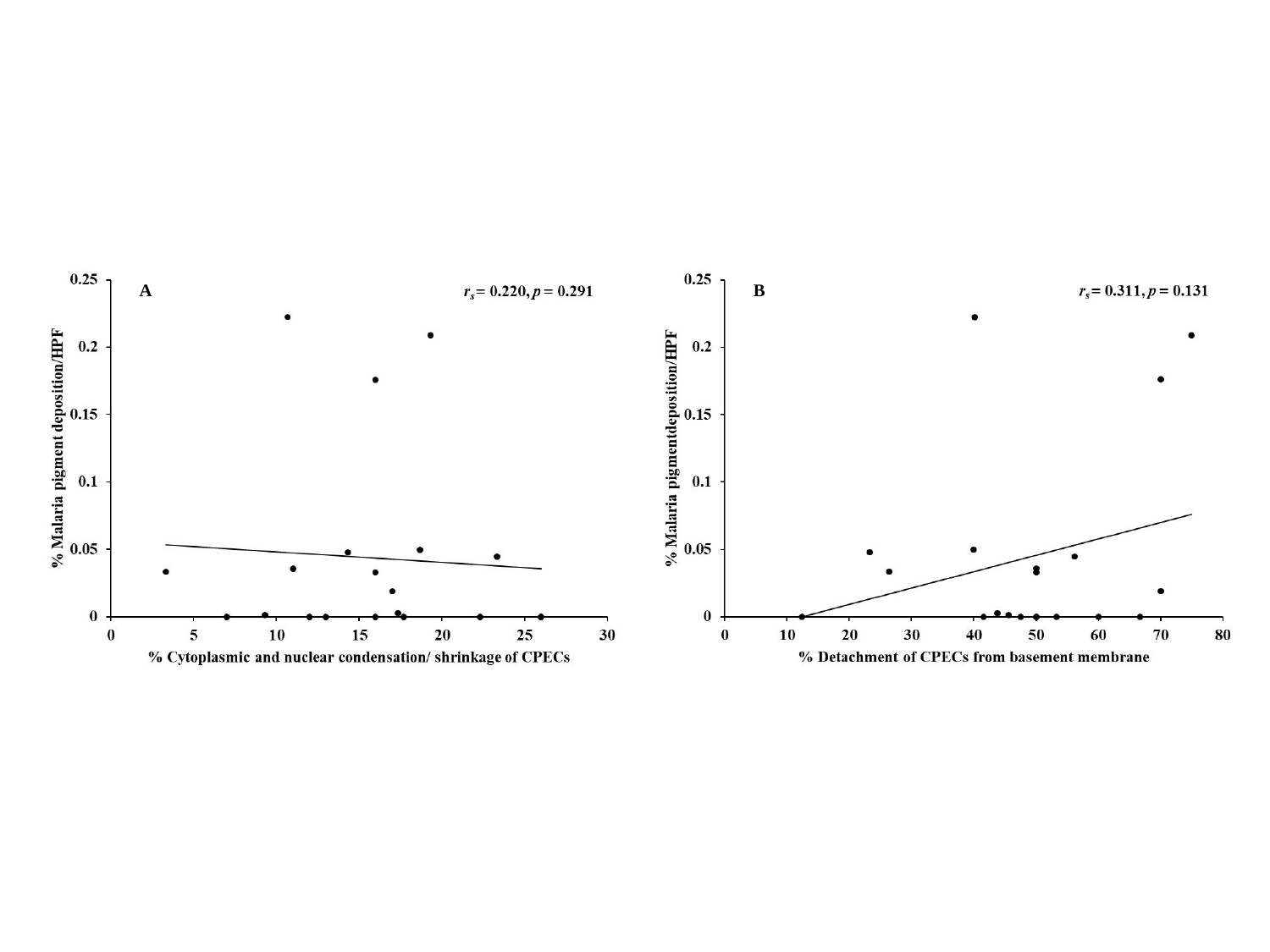

A
B

Supplement: Supplementary file 2 — Additional file 2: Figure S1. No pathological correlation between malaria pigment deposition and apoptosis of CPECs (A- cytoplasmic and nuclear condensation/shrinkage of CPECs, and B- detachment of CPECs from the basement membrane). [file 12936_2022_4044_MOESM2_ESM.ppt]

## Slide 1
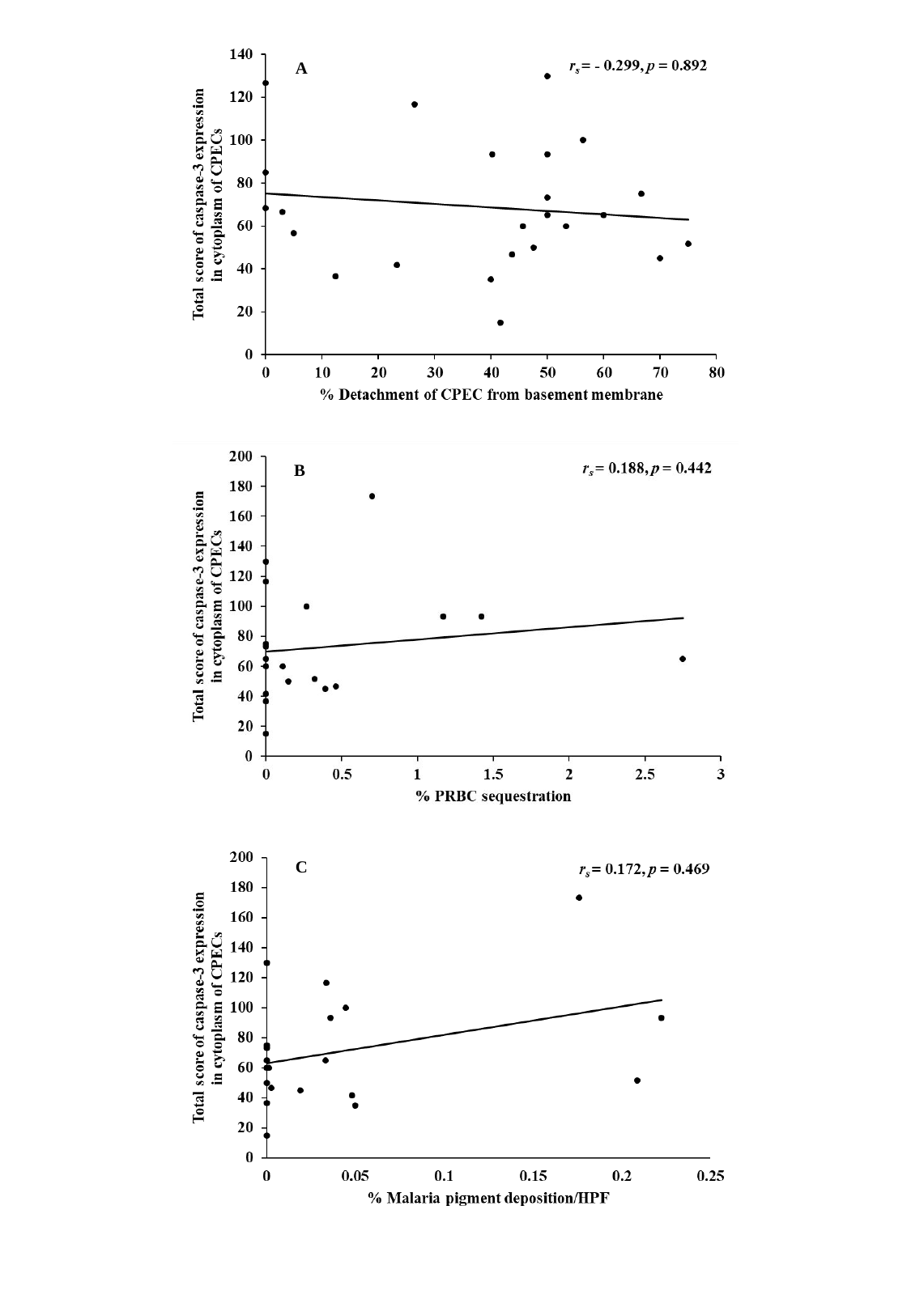

A
B
C

Supplement: Supplementary file 3 — Additional file 3: Figure S2. No correlation between caspase-3 and the degree of CPEC detachment from the basement membrane (A), PRBC sequestration (B), and presence of malaria pigments/haemozoin (C). [file 12936_2022_4044_MOESM3_ESM.ppt]

## Slide 1
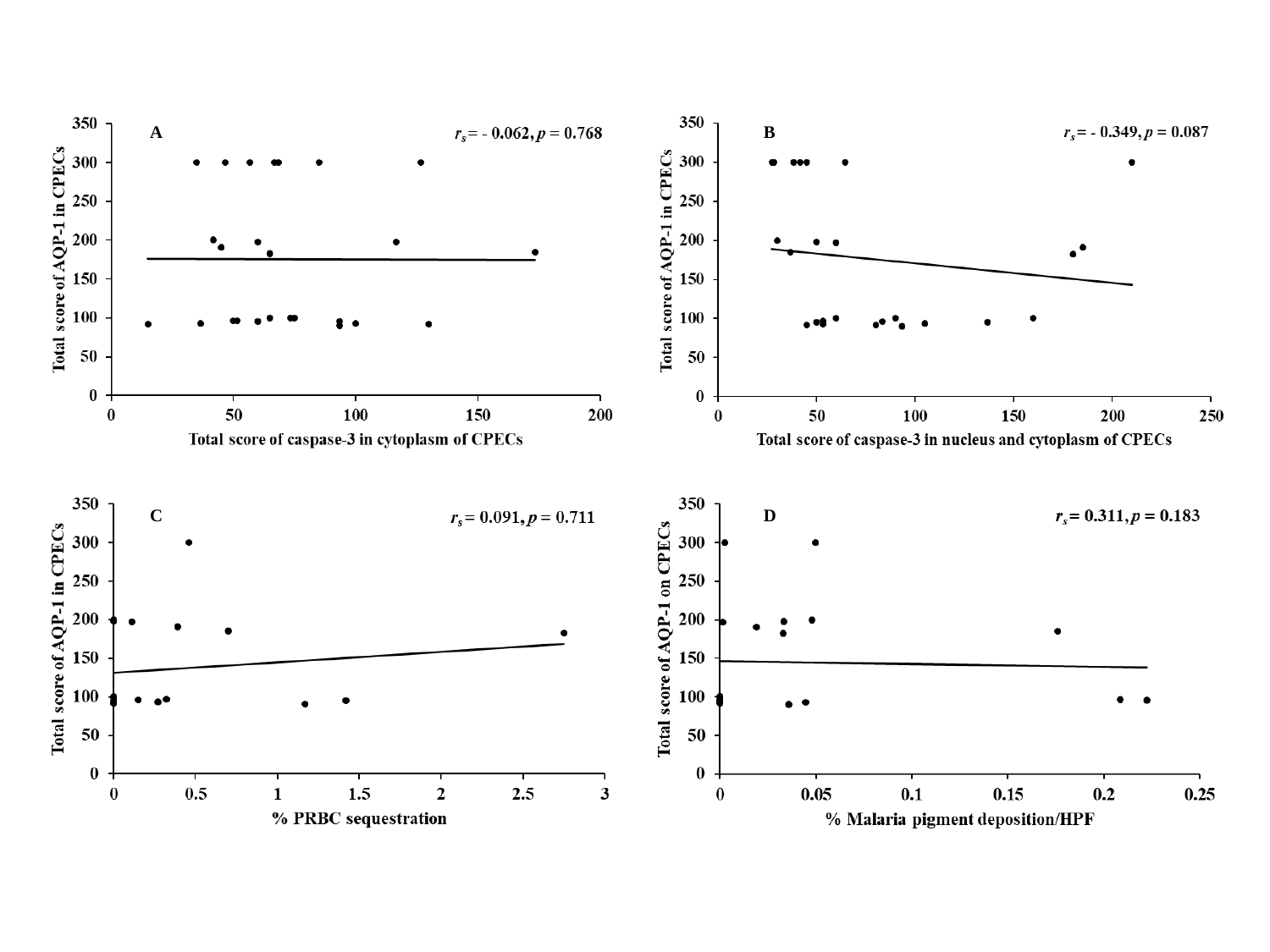

A
B
C
D

Supplement: Supplementary file 4 — Additional file 4: Figure S3. No correlation between AQP-1 expression in CPECs and caspase-3 expression in cytoplasm alone (A) and cytoplasm and nucleus (B), PRBC sequestration (C), and presence of malaria pigments/ haemozoin (D). [file 12936_2022_4044_MOESM4_ESM.ppt]
